# Supplementary material for: Electrothermal Modeling and Analysis of Polypyrrole-Coated Wearable E-Textiles
Source: Materials (Basel). 2021 Jan 24;14(3):550. doi: 10.3390/ma14030550 (PMC7865377; doi:10.3390/ma14030550)
Supplement: Supplementary file 1 [file materials-14-00550-s001.zip › supplementary/materials-1081297-supplementary.docx]

Supplementary Materials

Electrothermal Modeling and Analysis of Polypyrrole-Coated Wearable E-Textiles

Resistance Measurements

In the 2-probe method a current is applied to the outer contacts of a rectangular conductive sheet of known width and thickness and the voltage drop is measured between the inner electrodes using the following, Equation (S1):

| $\sigma=\left( \frac{z}{xy} \right)\frac{I}{V}$ | S1 |
| --- | --- |

where, σ is the conductivity in S/cm

z is the distance between the inner electrodes in cm

x is the sample width in cm

y is the film thickness in cm

I is the current supplied to the outer electrodes; V is the potential difference measured between the inner electrodes.


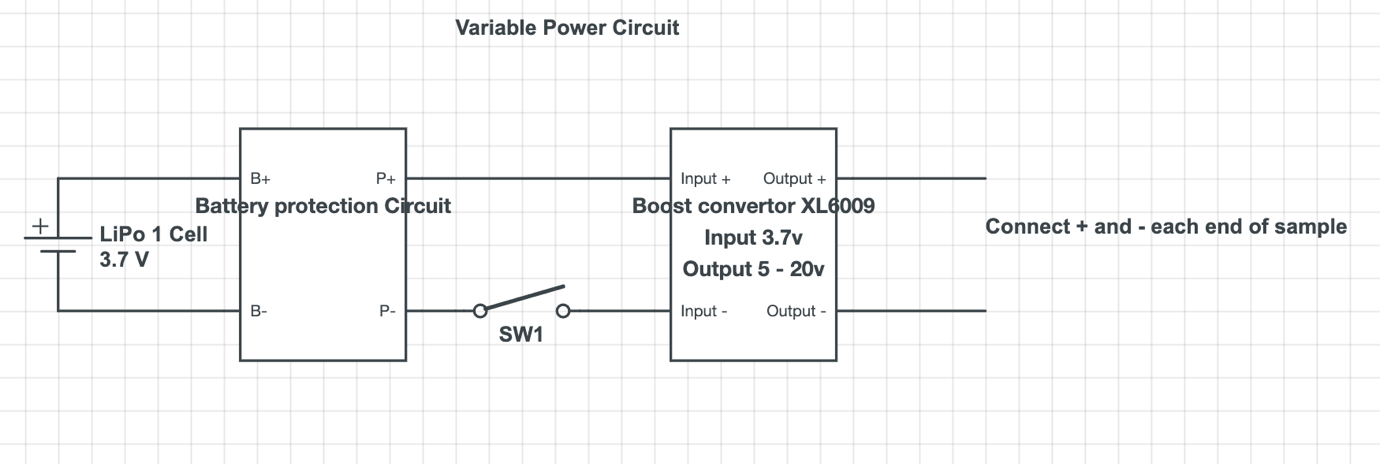


**Figure S1.** Schematic of the circuit created for heating analysis.

**Table S1.** Results of AQSA concentration analysis.

| **Sample** | **AQSQ (M)** | **Pyrrole (M)** | **Iron(III) Chloride Hexahydrate (M)** | **Resistance Average (Ohms)** |
| --- | --- | --- | --- | --- |
| $1$ | $0.01$ | $0.05$ | $0.05$ | $520$ |
| $2$ | $0.025$ | $0.05$ | $0.05$ | $245$ |
| $3$ | $0.05$ | $0.05$ | $0.05$ | $192$ |
| $4$ | $0.075$ | $0.05$ | $0.05$ | $124$ |
| $5$ | $0.1$ | $0.05$ | $0.05$ | $82$ |
| 6 | 0.125 | 0.05 | 0.05 | 132 |

**Table S2.** Resistance versus ferric chloride concentration.

| **Sample** | **AQSQ (M)** | **Pyrrole (M)** | **Iron(III) Chloride Hexahydrate (M)** | **Resistance Average (Ohms)** |
| --- | --- | --- | --- | --- |
| $1$ | $0.05$ | $0.05$ | $0.01$ | $212$ |
| $2$ | $0.05$ | $0.05$ | $0.025$ | $200$ |
| $3$ | $0.05$ | $0.05$ | $0.05$ | $192$ |
| $4$ | $0.05$ | $0.05$ | $0.075$ | $188$ |
| $5$ | $0.05$ | $0.05$ | $0.1$ | $187$ |
| $6$ | $0.05$ | $0.05$ | $0.125$ | $198$ |

**Table S3.** Results of pyrrole concentration analysis.

| **Sample** | **AQSQ (M)** | **Pyrrole (M)** | **Iron(III) Chloride Hexahydrate (M)** | **Resistance Average (Ohms)** |
| --- | --- | --- | --- | --- |
| $1$ | $0.05$ | $0.01$ | $0.05$ | $220$ |
| $2$ | $0.05$ | $0.02$ | $0.05$ | $217$ |
| $3$ | $0.05$ | $0.03$ | $0.05$ | $199$ |
| $4$ | $0.05$ | $0.04$ | $0.05$ | $194$ |
| $5$ | $0.05$ | $0.05$ | $0.05$ | $192$ |
| $6$ | $0.05$ | $0.06$ | $0.05$ | $196$ |
